# Stretching and Multicomponent Training to Functional Capacities of Older Women: A Randomized Study

**DOI:** 10.3390/ijerph19010027

**Published:** 2021-12-21

**Authors:** Andressa Crystine da Silva Sobrinho, Mariana Luciano de Almeida, Guilherme da Silva Rodrigues, Rodrigo Fenner Bertani, Joao Gabriel Ribeiro Lima, Carlos Roberto Bueno Junior

**Affiliations:** 1 School of Medicine of Ribeirao Preto, University of Sao Paulo (USP), Avenida Bandeirantes 3900, Ribeirao Preto 14049-900, SP, Brazil; andressa.sobrinho@usp.br (A.C.d.S.S.); guirodrigues@usp.br (G.d.S.R.); joao.ribeiro.lima@usp.br (J.G.R.L.); 2 College of Nursing of Ribeirao Preto, University of Sao Paulo (USP), Avenida Bandeirantes 3900, Ribeirao Preto 14049-900, SP, Brazil; ml.almeida@usp.br; 3 University of Franca (UNIFRAN), Avenida Dr. Armando de Sáles Oliveira 201, Franca 14404-600, SP, Brazil; rodrigo_fenner@yahoo.com

**Keywords:** exercise, older adults, functional performance, aging, physical fitness

## Abstract

Background and Purpose: The real benefits of stretching when used as training for the older adult population and for developing other physical capacities are still uncertain. Thus, the objective of the present work is to investigate the effects of stretching training combined with multi-component training on the physical capacities of physically inactive older women. Methods: Women aged 60 to 70 years were randomized into three groups: multicomponent training (MT), multicomponent training combined with flexibility training (CT), and control group (CG). Both training interventions were carried out for 14 weeks, with two weekly sessions. Participants were assessed for agility, muscle strength (sitting and standing and elbow flexion/extension), and cardiorespiratory fitness (6-min walk). Results and Discussion: Multicomponent training with flexibility presented a very large effect on the variables of strength, agility, and aerobic fitness, while multicomponent training had a medium effect on agility and a large and very large effect on muscle strength variables. This is the first study in the literature to analyze the effect of flexibility training, associated with multicomponent training, on other physical capacities. Conclusions: The results of the current study suggest that adding flexibility training to a multicomponent training program generates additional benefits to the development of other physical capacities.

## 1. Introduction

The aging process leads to a decline in biological, social, and functional capacities, including changes in the components of functional capacity, such as loss of flexibility, and decreased muscle strength, agility, cardiorespiratory capacity, and joint mobility [1]. These alterations can lead to a vicious cycle of physical inactivity and increase the risk for the development of chronic diseases, which can also culminate in injuries, falls, and a higher risk of mortality, demonstrating the need for interventions in this process [2,3,4].

The World Health Organization defends the benefits of physical activity for health (150 to 300 min per week, with emphasis on cardiorespiratory/strength, flexibility, and balance training), as it improves the cardiovascular, metabolic and immune, in addition to improving balance, strength, thus also acting as a tool for preventing falls and increasing independence. Furthermore, findings in the literature reveal that high levels of physical exercise practice increase the chances of living for more than 10 years without chronic comorbidities, cognitive damage, and lack of functional capacity. Thinking about strategies that further maximize these gains is essential for active aging [5,6]. 

The regular practice of physical exercise is an important intervention tool to reduce the dependence of older adults on the care of others to carry out their daily activities and to improve physical fitness [7]. Multicomponent training is a combination of three or more components of physical fitness in the same training session, which makes it a tool to improve physical performance and improve the overall health status of older people and have a high rate of adherence in this population [8,9]. Valenti et al. [8] demonstrated that multicomponent training promotes improvements in physically inactive older people’s physical and functional capacities. Although the American College of Sports Medicine and the American Heart Association guidelines for older adults indicate that a training program should present a multicomponent approach, most intervention studies offer training protocols that sometimes develop only muscular strength and/or aerobic capacity [9,10,11].

The most commonly used modality of physical exercise recommended for increasing flexibility is stretching, and the main health-related benefits promoted by increased flexibility are increased joint amplitude and improved posture. Muscle stretching also increases blood flow, endothelial function, capillarity, and vascular volume [12].

In the literature, it is suggested that flexibility is an important component for other capacities, as it plays an important role in stabilizing and maintaining joint movement/amplitude, and can contribute to postural balance and optimization of musculoskeletal function [13]. In many studies, flexibility/stretching exercises are applied only in the control group. Furthermore, older adults have a vast range of fitness. For example, for frail ones, flexibility should allow an adequate degree of motion when performing other movements [8,10,13]. Although there are recommendations from the World Health Organization (WHO) for applying flexibility training for health, flexibility is an often overlooked component in physical conditioning programs. When applied represents only 5–15% of session time [8,9].

Multicomponent training already improves several physical abilities of older people, and we want to find out if stretching can enhance/maximize these gains already existing in this type of exercise training. Despite considering the principle of specificity in exercise training, it is crucial to understand the interrelationships between physical fitness components. Thus, the present work aims to investigate, for the first time according to our knowledge, the effect of stretching training combined with multicomponent training on other physical abilities (muscle strength, aerobic endurance, and agility) in physically inactive older women. We hypothesized that interventions with exercises to improve flexibility contribute to the mechanical properties of the connective and muscle tissue, enabling functional improvements to the musculature and, thus, contributing to the development of other physical abilities, since the literature shows that more flexible people have more agility and improve their walk, contributing to an increase in physical performance [8,9].

## 2. Materials and Methods

The research and informed consent form were submitted to and approved by the Research Ethics Committee with Human Beings of the School of Physical Education and Sport of Ribeirão Preto, University of São Paulo (CAAE: 63681517.3.0000.5659) and registered in the Brazilian Registry of Clinical Trials (REBEC: RBR-8hqwmx).

### 2.1. Study Design

Participants were recruited through advertising in local media and on social networks. Before the first assessment, the participants attended a lecture to present the project and immediately afterwards signed the free and informed consent form. After this stage, the participants were divided into two groups, separated according to ages from 60 to 65 years and 66 to 70 years, for subsequent blind randomization into three groups: multicomponent training (MT), multicomponent training combined with flexibility training (CT), and control group (CG), ensuring homogeneity between groups in relation to age. 

**Figure 1:**
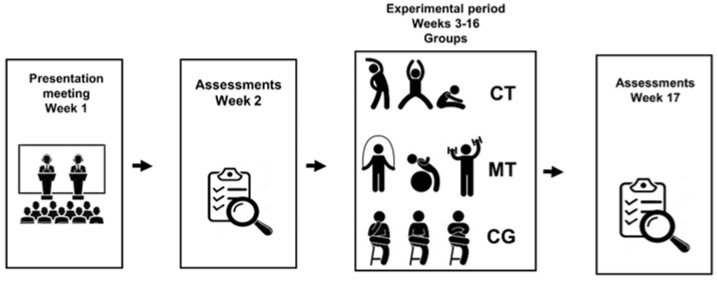
Study design. Abbreviations: CT; combined training; CG, control group; MT, multicomponent training.

In the power analysis, 40 participants per group would be necessary to detect a difference between means of 8.5 cm for the primary outcome (frontal plane asymmetry or sagittal plane asymmetry), with the alpha error probability set at 0.05 and power adjusted to 0.8. We used G Power 3.1.9.7 (Kiel University, Kiel, Germany) to calculate the power. Pre- and post-trial evaluations were carried out, which lasted 14 weeks (Figure 1).

### 2.2. Participants 

The inclusion criteria adopted were: being female aged 60 to 70 years, having a medical certificate of release to practice physical activity, and being physically inactive according to the scores of the Modified Baecke Questionnaire for Older Adults (MBQI < 9.11). The exclusion criteria were having diseases and/or functional limitations (motor, auditory, and visual deficits) that would prevent the performance of tests and/or the proposed physical training, and absences from more than 25% of physical training sessions.

### 2.3. Interventions

The control group did not participate in any intervention or contact the evaluators during the training period. The contact was made only for pre and post evaluations.

#### 2.3.1. Multicomponent Training

The multicomponent training consisted of two classes per week of 90 min each, divided into a 15-min warm-up, consisting of balance exercises, motor coordination and games, 35 min of muscular strength, 35 min of aerobic activities, and five final minutes of relaxation, in order to develop the coordinating and conditioning motor capacities [14].

The intensities of the training sessions were prescribed and monitored using the Borg scale of subjective perception of effort adapted by Foster [15] (SPE), on which the values progress from 0 to 10, with 0 corresponding to no effort (rest) and 10 to the maximum effort (exhaustive). Around 12 exercises were chosen during each training session, arranged in a circuit format on a sports court. The exercises chosen were always multi-joint and encompassed the large body muscles. Materials such as mats, dumbbells, bars, step washers, medicine balls, tension elastics, and anklets increased the training load. The intensities of the training sessions were progressively prescribed, as follows: weeks 1 to 2:3 to 4; weeks 3 to 5:4 to 6; weeks 6 to 8:6 to 7; weeks 9 to 11:7 to 8; weeks 11 to 14:8 to 10, representing a moderate to high intensity of physical exercise.16 To measure the internal training loads (ITL), the training impulse (TRIMP) was used—the product of the multiplication of the SPE by the duration of the session in minutes (SPE × duration of the session), represented in arbitrary units (au). In addition to the ITL quantification, the monotony index (MI) was calculated according to Foster [15], which is used to determine the internal load variability during a given training period and is the result of the ratio between the mean daily load and the standard deviation (SD) of these values. Therefore, the increase in monotony represents low variation in the ITL pattern.

#### 2.3.2. Individualized Flexibility Training

Flexibility was trained through the active stretching method with accessories, according to the protocol proposed by Nelson and Kokkonen (2007) [16], which follow the recommendations of the American College of Sports Medicine (2019) [17], regarding volume and intensity [18]. The participants were separated into groups to perform stretching exercises aimed at postural alterations typical of aging (hip flexor muscles, spine extensors, elevators, and scapular protectors), focusing on individual needs, identified after the postural analysis test. The exercise protocol was directed to each postural compensation strategy and stratified into four levels of exercise complexity, with a new complexity added every four weeks. The intensity and volume protocol was divided into four levels, with progression of stretching time and painful perception measured by the pain scale [19]. Training was performed twice a week (Table S1).

### 2.4. Evaluations 

#### 2.4.1. Evaluation of Sample Characterization 

The characterization of the sample was performed using a questionnaire prepared by the researchers for the analysis of demographic and health data. Systolic (SBP) and diastolic (DBP) blood pressures were measured using a previously calibrated automatic digital blood pressure gauge (OMRON^®^, Jundiaí, Brazil, model HEM-7113, SBH, 2010), and anthropometric measures were taken (body mass, height, waist circumference, and body mass index). The level of physical activity of the participants was measured subjectively using the MBQI17 in conjunction with a triaxial accelerometer (GT3X-BT by ActiGraph, Pensacola, FL, USA)—the participants were instructed to wear the device for one week, and four days of the week and one day of the weekend were considered for the calculation [20]. The intensities of the activities were as stipulated by Freedson et al. [21]. 

#### 2.4.2. Motor Evaluations

The Senior Fitness Test (SCT) was used to evaluate functional capacity and obtain normative values [22]. This battery includes tests that evaluate the strength of the upper (elbow flexion and extension) and lower limbs (stand and sit in the chair), flexibility of the upper (reach behind the back) and lower limbs (sit and reach), and aerobic endurance (6-min walk (6 MW)) according to the age group. To evaluate the agility capacity, the adapted AAHPERD battery protocol was used—agility test/dynamic balance (two cones positioned 1.50 m behind and 1.80 m on each side) [23].

### 2.5. Statistical Analysis

The data obtained were organized in a double entry database, using Excel^®^, version 2013 (Microsoft, Redmond, DC, USA) and the SPSS^®^ statistical program, version 20.0 (International Business Machines Corporation-IBM, Armonk, New York, NY, USA). Data are presented as mean and standard deviation. To evaluate the normality of the data, the Kolmogorov-Smirnov test was used, and the variances were analyzed by the Levene test. The analysis of training comparisons was performed using the ANOVA two way statistical method of repeated measures, with Tukey’s post-hoc. The effect size was calculated by Cohen’s d, with values from 0.5 to 0.79 representing medium effect, values between 0.8 to 1.3 large effect, and greater than 1.3 very large effect—numbers below 0.5 were considered as a small effect [24,25]. The Student’s t test was used to verify the differences between the means of the intensity control measures and internal training load (Borg, TRIMP, and monotony index). The level of alpha significance adopted was 5%.

### 2.6. Data Availability

The data associated with the paper are not publicly available but are available from the corresponding author on reasonable request.

## 3. Results

The sample was characterized in a previous study (manuscript under review). The analyses included 43 women from the CT group, 52 from the MT group, and 47 from the CG group. There were no statistical differences between the groups in mean age (63.4 ± 5.6 years, considering all groups) and height (1.58 ± 0.08 m). Body mass decreased in the groups that trained and increased in the CG group in relation to the baseline moment. However, considering the body mass index, only the CG group presented an increase, with a large effect size. The groups that trained showed improvement in the level of physical activity assessed by the MBQI and by accelerometry, with the MT presenting a moderate effect size, the CT a very large effect size, and the CG a large effect size only in the accelerometry variable. 

Table 1 shows a moderate effect size for the internal training load variable (TRIMP) and a large effect size for the subjective perception of effort (BORG) among the groups who trained. Monotony, on the other hand, presents a statistical difference between the groups that trained, but with a small effect size. In the three variables, higher values were obtained by the CT group.

Table 2 presents the results of the physical and functional capacities. It was possible to observe a time-group interaction in the variables sitting and standing (F = 38.81; *p <* 0.001), elbow flexion and extension (F = 117.10; *p <* 0.001), 6-min walk (F = 6.67; *p* = 0.002), and agility (F = 5.45; *p* = 0.005). In these variables there was an improvement from the pre to the post-intervention moment in the two training groups, except for the six-minute walk variable, for which there was improvement only in the CT group.

When considering the effect size in Table 2, it can be observed that the CT group presents four variables with an effect size ≥ 0.50, all of which refer to positive baseline results and very large effect sizes (≥1.30). The MT group presents four variables with an effect size ≥ 0.50, all of which refer to positive baseline results. Of these, two variables have a medium effect size, one a large effect size, and one variable has a very large effect size. The CG group presents two variables with an effect size ≥ 0.50, with one positive result (mean effect size) and one negative result referring to the baseline (large effect size).

## 4. Discussion

The current study investigated the effects of 14 weeks of multi-component training with or without a flexibility training protocol on the development of other physical and functional capacities of women aged 60 to 70 years. The findings of the current study demonstrate the importance of including stretching exercises in physical training programs, which is associated with improvement in the parameters impaired by aging, contributing to improvement in other physical capacities. Flexibility has a direct relationship with the improvement in physical fitness and is considered an indicator of health [26]. In the present study, the multicomponent training group with an emphasis on flexibility demonstrated better results in physical parameters, with greater effect sizes. In addition, it is interesting to note that the 6-min walk results only improved in the CT group, which obtained better results considering the larger effect size in the agility and strength tests. The control group presented worsening agility values, demonstrating that 14 weeks was sufficient for the occurrence of declines in older adults who did not train during the intervention.

In the MT group, improvement was observed in all physical capacities, except for cardiorespiratory capacity. The literature suggests that the development of flexibility in a strength training program occurs because the exercises stimulate the maximum range of joints [27,28]. It has also been shown that multicomponent training is able to generate small gains in the flexibility capacity, as well as development of other physical capacities, as demonstrated in the study of Cadore et al. [29], in which 16 weeks of training for older adults resulted in improvements in some physical capacities and maintenance of adequate levels of flexibility for daily activities.

In the study by Kang et al. [30], older people also participated in a multicomponent training program and showed improvements in health-related physical fitness components—strength, cardiorespiratory capacity, flexibility, and body composition [31,32]. Our study also demonstrated improvement in muscle strength and agility due to multicomponent training, particularly in the CT group. 

Therefore, in relation to physical capacities, the groups that trained demonstrated improvements, highlighting the CT group. According to Lyakh et al. [33], low levels of flexibility can result in little assimilation of motor skills, and restricted levels of strength, speed, coordination, and agility. According to Joho et al. [34], a high level of flexibility is of fundamental importance at any age.

The most commonly cited capacity associated with flexibility in the literature is strength, mainly explosive strength (power) and maximum strength [35]. However, a common view of flexibility associated with strength is that stretching before a strength training session may reduce performance, and after the training session, may increase the risk of injury [35,36]. These data are from adults and athletes. We have seen an approach more focused on analgesia, relaxation, and mobility gain with flexibility in older populations [8,13]. In contrast, our study demonstrated that muscle strength can be increased with the development of flexibility capacity, which may indicate the importance of including flexibility training as well as strength training for this population [37,38]. The systematic review of Higgs and Winte [39] showed that flexibility training has a chronic beneficial effect on strength capacity, and a recommendation should be maintained to include flexibility exercise routines in parallel with strength training, as a way to obtain better strength gains. Subsequently, other studies found the same results [36], corroborating with our CT group, which resulted in greater strength gains when compared to the MT group.

The results of the current study demonstrated that the walk test, which evaluates cardiorespiratory fitness, showed significant improvement only in the CT group. This can be explained by the fact that adequate levels of flexibility contribute to the step amplitude, by means of elastic energy, enabling an increase in the mechanical efficiency of the muscles, and subsequent energy savings during efforts at different intensities [40]. The study of Jadczak et al. [41], verified the effectiveness of exercise interventions, such as multicomponent and functional, on the physical function of older adults, and demonstrated that interventions that encompassed flexibility, balance, and other physical capacities resulted in satisfactory improvement in the walking test. Studies that analyzed the influence of flexibility capacity directly on walking performance are still scarce. Sekendiz et al. [42], suggested that continuous flexibility training may result in a decrease in contact between the actin and myosin filaments, leading to greater recruitment of inactive muscle fibers as a way of compensation, improving walking performance, as seen in the CT group in our results. 

Improvements in agility were observed in both groups that trained. However, the CT group demonstrated a larger effect size than the other groups. The test performed in our study, in addition to assessing agility, also assesses dynamic balance. The literature points out that training methods to gain flexibility can maintain or slightly increase levels of agility [43]. However, there are still uncertainties about this relationship. The study of Donath, van Dieën and Faude [44], demonstrates that the agility capacity is a sum of strength, speed, and balance. Improvement in dynamic balance/agility through flexibility training can contribute to the reduction in falls, which is very common in this population [43,44].

Carneiro et al. [45], compared the levels of strength, flexibility, and cardiorespiratory fitness of independent and physically active older people with those of physically inactive older people, and found significant correlations between flexibility and levels of physical activity. The authors also observed that physically active older people, in addition to living autonomously, had higher levels of general flexibility, higher walking speed, better cardiorespiratory fitness, and better reported quality of life, when compared to physically inactive older people. Our results corroborate these findings, since flexibility training resulted in better values in other physical capacities, which could contribute to autonomy and quality of life. In this context, it is interesting to emphasize the importance of inclusion of flexibility exercises in physical training programs in order to improve physical and functional fitness.

### Limitation

As a limitation of the study, we suggest the intervention time as some studies point to more prolonged interventions for the physical capacities studied. As a strong point, we highlight that we used accelerometry to analyze the level of physical activity throughout the week—this is an important variable to be controlled in our study.

## 5. Conclusions

Multicomponent training with flexibility presented a very large effect on the variables of strength, agility, and aerobic fitness, while multicomponent training had a medium effect on agility and aerobic fitness and a large/very large effect on muscle strength. Thus, we can conclude that multicomponent training with flexibility enhances the development of the studied physical capacities.

## Figures and Tables

**Table 1:** Variables used to control training stimuli.

	MT (n = 52)	CT (n = 4 3)	d-Cohen
Trimp (au)	667.0 ± 42.9	693.9 ± 58.1 *	**0.533**
Borg (au)	7.3 ± 0.4	7.7 ± 0.5 *	**0.889**
Monotony (au)	4.4 ± 1.0	5.3 ± 3.1 *	0.439

**Note:** CT; combined training; MT, multicomponent training; au: arbitrary unit. *: *p* < 0.05 for differences between groups (Test t-Student). Bold: effect size ≥ 0.5.

**Table 2:** Assessments of physical and functional capabilities through motor tests.

	MT (n = 52)			CT (n = 43)			CG (n = 47)		
	Pre	Post	Effect Size	Pre	Post	Effect Size	Pre	Post	Effect Size
Sitting and standing (rep) ^†^	12 ± 4	18.1 ± 4.4 *	**1.452**	13.6 ± 4.4	25.1 ± 5.4 *^,$^	**2.347**	12.4 ± 5.1	11.3 ± 5.4 ^#,$^	0.210
Elbow flexion and extension (rep) ^†^	14.6 ± 4.4	20 ± 4.7 *	**1.187**	16.7 ± 3.8	27.2 ± 4.3 *^,$^	**2.593**	13.7 ± 4.7	14.8 ± 3.5 ^#,$^	0.268
Six-minute walk (m) ^†^	522.2 ± 70.7	594.7 ± 119.6	**0.762**	526.8 ± 56.9	716.9 ± 68.2 *^,$^	**3.039**	520.9 ± 66.7	558.8 ± 78.6 ^#^	**0.522**
Agility (s) ^†^	26.9 ± 3.2	25 ± 3.4 *	**0.576**	27.4 ± 4	19.6 ± 4 *^,$^	**1.950**	25.2 ± 3.4	27.6 ± 2.4 ^#,$^	**0.828**

**Note**: CT; combined training; CG, control group; MT, multicomponent training. ^†^: interaction between time and group (*p* < 0.05); *: *p <* 0.05 in relation to the pre-intervention moment in the same group; ^#^: *p <* 0.05 in relation to the TMF at the same time; ^$^: *p <* 0.05 in relation to the TM at the same time. Bold: effect size ≥ 0.50.

## Data Availability

The data presented in this study are available on request from the corresponding author. The data are not publicly available due to consent provided by participants.

## Supplementary Materials

The following are available online at https://www.mdpi.com/article/10.3390/ijerph19010027/s1, Table S1: Flexibility Training Protocol Adopted in the Intervention.

## References

[1] Rebelo-Marques A., Lages A.S., Andrade R., Ribeiro C.F., Pinto A.M., Carrilho F., Mendes J.E. (2018). Aging hallmarks: The benefits of physical exercise. Front. Endocrinol. (Lausanne).

[2] Labra C., Guimaraes P.C., Maseda A., Lorenzo T., Millán C.J.C. (2015). Effects of physical exercise interventions in frail older adults: A systematic review of randomized controlled trials. BMC Geriatr..

[3] Huisingh S.M., Wroblewski K., Kocherginsky M., Huang E., Dale W., Waite L., Schumm L.P. (2018). The relationship between physical activity and frailty among U.S. older adults based on hourly accelerometry data. J. Gerontol. A Biol. Sci. Med. Sci..

[4] Portegijs E., Karavirta L., Saajanaho M., Rantalainen T., Rantanen T. (2019). Assessing physical performance and physical activity in large population-based aging studies: Home-based assessments or visits to the research center?. BMC Public Health.

[5] Thandi M.K.G., Phinney A., Oliffe J.L., Wong S., McKay H., Sims-Gould J., Sahota S. (2018). Engaging older men in physical activity: Implications for health promotion practice. Am. J. Men’s Health.

[6] WHO (2010). Global Recommendations on Physical Activity for Health.

[7] Bangsbo J., Blackwell J., Boraxbekk C.J., Boraxbekk C.J., Caserotti P., Dela F., Vina J. (2019). Copenhagen consensus statement 2019: Physical Activity and Ageing. Br. J. Sports Med..

[8] Valenti G., Bonomi A.G., Westerterp K.R. (2016). Multicomponent fitness training improves walking economy in older adults. Med. Sci. Sports Exerc..

[9] Turunen K., Salpakoski A., Edgren J., Törmäkangas T., Arkela M., Kallinen M., Sipilä S. (2017). Physical activity acter a hip fracture: Effect of a multicomponent home-based rehabilitation program-a secondary analysis of a randomized controlled trial. Arch. Phys. Med. Rehabil..

[10] Machado O.A.S., Lima W.P., Souza B.V., Gianolla F., Killian L.F., Machado G.A.C., Gorjão R. (2019). Comparison of functional capability, flexibility, strength and quality of life in aged women engaged in resistance exercise, weight-bearing training or hydro-gymnastics. J. Sport Sci..

[11] Caldas L.R.R., Albuquerque M.R., Lopes E., Moreira A.C., Ribeiro A.Q., Carneiro-Júnior M.A. (2020). Multicomponent physical training increases strength, agility and dynamic balance in middle-aged women. Revista Brasileira de Fisiologia do Exercí Cio.

[12] Medeiros D.M., Lima C.S. (2017). Influence of chronic stretching on muscle performance: Systematic review. Hum. Mov. Sci..

[13] Zambon T.B., Gonelli P.R.G., Gonçalves R.D., Borges B.L.A., Montebelo M.D.C. (2015). Análise comparativa da flexibilidade de mulheres idosas ativas e não ativas. Acta Fisiatr..

[14] Trapé A.A., Lizzi E.A.D.S., Gonçalves T.C.P., Rodrigues J.A.L., Tavares S.S., Lacchini R., Bueno Junior C.R. (2017). Effect of multicomponent training on blood pressure, nitric oxide, redox status, and physical fitness in older adult women: Influence of endothelial nitric oxide synthase (NOS3) Haplotypes. Oxid. Med. Cell. Longev..

[15] Foster C. (1998). Monitoring Training in Athletes with Reference to Overtraining Syndrome. Med. Sci. Sports Exerc..

[16] Borg G.A., Noble B.J. (1974). Perceived exertion. Exerc. Sport Sci. Rev..

[17] Nelson A., Kokkonen J. (2007). Stretching Anatomy.

[18] Patel A.V., Friedenreich C.M., Moore S.C., Hayes S.C., Silver J.K., Campbell K.L., Matthews C.E. (2019). American college of sports medicine roundtable report on physical activity, sedentary behavior, and cancer prevention and control. Med. Sci. Sports Exerc..

[19] Thong I.S.K., Jensen M.P., Miró J., Tan G. (2018). The validity of pain intensity measures: What do the NRS, VAS, VRS, and FPS-R measure?. Scand. J. Pain.

[20] Sobrinho A.C.S., Almeida M.L., Silva V.R.R., Wiggers E., Bueno Júnior C.R. (2019). Relationship between cognitive performance, sedentary behavior and physical activity level of brazilian older people women. Alzheimers Dement..

[21] Freedson P.S., Melanson E., Sirard J. (1998). Calibration of the computer science and applications, Inc. accelerometer. Med. Sci. Sports Exerc..

[22] Riklli R.E., Jones C.J. (1999). Development and validation of a functional fitness test for community-residing older adults. J. Aging Phys. Act..

[23] Osness W.H., Adrian M., Clark B., Hoeger W., Raab D., Wiswell R. (1990). Functional Fitness Assessment for Adults over 60 Years. https://www.scienceopen.com/document?vid=24e042cb-a996-4b68-a239-64b397f9dd53.

[24] Lee D.K. (2016). Alternatives to P value: Confidence interval and effect size. Korean J. Anesth..

[25] Leppink J., O’Sullivan P., Winston K. (2016). Effect size-large, medium, and small. Perspect. Med. Educ..

[26] Bucht H., Donath L. (2019). Sauna Yoga Superiorly Improves flexibility, strength, and balance: A two-armed randomized controlled trial in healthy older adults. Int. J. Environ. Res. Public Health.

[27] Jung K.S., In T.S., Cho H.Y. (2017). Effects of sit-to-stand training combined with transcutaneous electrical stimulation on spastiitly, muscle strength and balance ability in patients with stroke: A randomized controlled study. Gait Posture.

[28] Leite T.B., Costa P.B., Leite R.D., Novaes J.S., Fleck S.J., Simão R. (2017). Effects of different number of sets of resistance training on flexibility. Int. J. Exerc. Sci..

[29] Cadore E.L., Sáez de Asteasu M.L., Izquierdo M. (2019). Multicomponent exercise and the hallmarks of frailty: Considerations on cognitive impairment and acute hospitalization. Exp. Gerontol..

[30] Kang S., Hwang S., Klein A.B., Kim S.H. (2015). Multicomponent exercise for physical fitness of community-dwelling older people women. J. Phys. Ther. Sci..

[31] Lima C.Z.L. (2018). Efeitos da periodização linear versus ondulatório diária no treinamento de força sobre a flexibilidade. Brazilian J. Exerc. Physiol. Prescr. (RBPFEX).

[32] Simão R., Lemos A., Salles B., Leite T., Oliveira E., Rhea M., Reis V.M. (2011). The influence of strength, flexibility, and simultaneous training on flexibility and strength gains. J. Strength Cond. Res..

[33] Lyakh V., Mikołajec K., Bujas P., Litkowycz R. (2014). Review of platonov’s “sports training periodization. general theory and its practical application”-kiev: Olympic literature, 2013. J. Hum. Kinet..

[34] Joho K., Abe T., Seol J., Fujii Y., Fujii K., Okura T. (2017). Examining physical functioning in older adults: A comparison of two stretching practice methods. Innov. Aging.

[35] Fjerstad B.M., Hammer R.L., Hammer A.M., Connolly G., Lomond K.V., O’Connor P. (2018). Comparison of two static stretching Procedures on Hip Adductor Flexibility and Strength. Int. J. Exerc. Sci..

[36] Kaya F. (2018). Positive Effects of proprioceptive neuromuscular facilitation stretching on sports performance: A review. J. Educ. Train Stud..

[37] Ball S., Gammon R., Kelly P.J., Cheng A.L., Chertoff K., Kaume L., Abreu E.L., Brotto M. (2013). Outcomes of stay strong, stay healthy in community settings. J. Aging Health..

[38] Syed-Abdul M.M., Ball S.D. (2021). Muscle activation in older females after a community-based resistance training program: A pilot study. Reports.

[39] Higgs F., Winter S.L. (2009). The effect of a four-week proprioceptive neuromuscular facilitation stretching program on isokinetic torque production. J. Strength Cond. Res..

[40] Brooks J.H., Fuller C.W., Kemp S.P., Reddin D.B. (2006). Incidence, risk, and prevention of hamstring muscle injuries in professional rugby union. Am. J. Sports Med..

[41] Jadczak A.D., Makwana N., Luscombe-Marsh N., Visvanathan R., Schultz T.J. (2018). Effectiveness of exercise interventions on physical function in community-dwelling frail older people: An umbrella review of systematic reviews. JBI Database Syst. Rev. Implement Rep..

[42] Sekendiz B., Cuğ M., Korkusuz F. (2010). Effects of swiss-ball core strength training on strength, endurance, flexibility, and balance in sedentary. J. Strength Cond. Res..

[43] Richman E.D., Tyo B.M., Nicks C.R. (2019). Combined effects of self-myofascial release and dynamic stretching on range of motion, jump, sprint, and agility performance. J. Strength Cond. Res..

[44] Donath L., van Dieën J., Faude O. (2016). Exercise-based fall prevention in the older people: What about agility?. Sports Med..

[45] Carneiro N.H., Ribeiro A.S., Nascimento M.A., Gobbo L.A., Schoenfeld B.J., Júnior A.A., Cyrino E.S. (2015). Effects of different resistance training frequencies on flexibility in older women. Clin. Interv. Aging.

