# Supplementary material for: Stretching and Multicomponent Training to Functional Capacities of Older Women: A Randomized Study
Source: Int J Environ Res Public Health. 2021 Dec 21;19(1):27. doi: 10.3390/ijerph19010027 (PMC8751009; doi:10.3390/ijerph19010027)
Supplement: Supplementary file 1 [file ijerph-19-00027-s001.zip › ijerph-1451429-supplementary.pdf]

Table S1. Flexibility Training Protocol Adopted in the Intervention.

	**Level 1**	**Level 2**	**Level 3**	**Level 4**
Week of intervention	1-2	3-6	7-10	11-14
Duration of session	20″	30″	40″	50″
Time under tension	10″	15″	20″	25″
Interval between series	10″	15″	20″	25″
Series per exercise	2	3	4	5
Pain level*	1 a 3	2 a 4	4 a 6	6 a 8
Exercises per body region$	2	3	3	4
Weekly dose#	2400″	3600″	4800″	6000″

Note: *, numeric visual/verbal scale of pain from 0 to 10; $, 8 body regions were worked in each individual-initial evaluations of each participant were considered for choice of these regions; #, weekly dose (seconds) = duration of session (min) * 2 (sessions/week) * 60 (seconds/minute).
